# The compositional homogeneity of the metal particle during vapor–liquid–solid growth of nanowires

**DOI:** 10.1038/s41598-020-67618-x

**Published:** 2020-07-06

**Authors:** Jonas Johansson, Niels Chr. Overgaard, Martin H. Magnusson

**Affiliations:** 1 Solid State Physics and NanoLund, Lund University, Box 118, 221 00 Lund, Sweden; 2 Centre for Mathematical Sciences, Lund University, Box 118, 221 00 Lund, Sweden

**Keywords:** Materials science, Nanoscale materials, Nanowires

## Abstract

The vapor–liquid–solid (VLS) mechanism is probably the most versatile method to fabricate semiconductor nanowires and several investigations assume a compositionally homogeneous catalyst particle. In this investigation we address the compositional homogeneity of the catalyst particle during growth of nanowires. Using diffusion calculations, we show that the particle is indeed homogeneous during VLS growth, but can have a strong concentration gradient during vapor–solid–solid growth, that is, growth with a solid particle. We also show that the response to a concentration change is extremely fast, meaning that if the concentration at the surface of the particle changes, the entire particle reaches this new concentration effectively instantaneously.

## Introduction

The vapor–liquid–solid (VLS) growth mechanism was first proposed by Wagner and Ellis in 1964^1^ to explain Si whisker growth. Later, in the 1970′s, Givargizov contributed significantly to the understanding of the VLS mechanism. He investigated the nanowire diameter dependence of the growth rate, both in terms of the Gibbs–Thomson effect and in terms of side facet diffusion^2^. In the early 2000′s whisker growth had a renaissance, to a great extent spurred by Hiruma’s research in the 1990’s^3^, and the whiskers grown since then are often orders of magnitude thinner and are more commonly referred to as nanowires. Such nanowires, made of III–V semiconductors and fabricated using liquid metal alloys (often gold- or group III-based) as catalyst particles, are being widely investigated in several application areas, where photovoltaics^4^ and solid state lighting^5^ are two major ones.

These and other applications demand highly controllable fabrication, which requires a thorough understanding of the nanowire growth process. This fact, often spurred by scientific curiosity, has motivated many efforts in developing theories for VLS growth, see for instance^6^ for an overview. Many of these theories include the composition of the metal catalyst particle^7–9^, which is often explicitly^10^, or implicitly, assumed to be homogeneous. This is summarily justified by the notion that the diffusion through a small liquid drop should be sufficiently fast to make the assumption of a homogeneous composition valid. In most cases, as we will show, this is indeed true and the assumption is valid. However, a quantitative analysis of the compositional homogeneity of the metal particle during VLS growth has until now been missing.

Warranted by the importance of this knowledge, we here introduce a measure for this homogeneity in the form of a dimensionless number, which turns out to be related to the Damköhler number and to the mass transfer Biot number. We calculate this number and show that in typical cases of VLS growth, it is indeed safe to assume that the catalyst particle is homogeneous. Moreover, we estimate the time it takes to homogenize a liquid catalyst particle. More precisely, we calculate the time it takes for a particle with zero initial concentration to reach a concentration at the solid–liquid growth interface that approaches the concentration at the surface, that is, at the liquid–vapor interface. Based on our results, we conclude that diffusion through the liquid catalyst particle is rarely the limiting factor in VLS growth of nanowires.

## Catalyst particle homogeneity

We start our analysis by introducing a measure for the relative maximum concentration difference in the catalyst particle during steady state nanowire growth. This measure also describes the compositional homogeneity of the particle. Here we assume that the growth proceeds continuously with the steady state axial growth rate, which thus sets the rate that atoms from the liquid incorporate into the solid. The measure is a dimensionless number, $\chi$, that we define as

1 $$ \chi = \frac{{c_{{\max}} - c_{{\min}} }}{{c_{{\max}} }}, $$

where $c_{{\max}}$ and $c_{{\min}}$ are the maximum and minimum concentrations of nanowire-constituting atoms in the particle, respectively. From this definition it is clear that $0 \le \chi \le 1$, where $\chi = 0$ indicates a compositionally homogeneous particle and $\chi = 1$ accounts for the maximum attainable concentration gradient through the particle.

**Figure 1:**
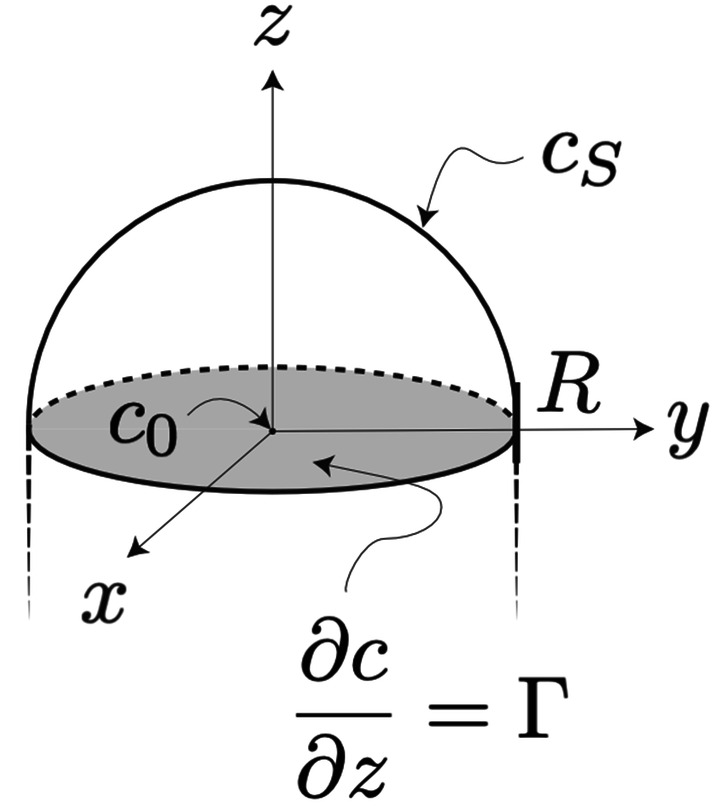
Schematic illustration of the geometry of the hemispherical catalyst particle with the coordinate system and boundary conditions indicated. The notation for the different parts of the domain are: ${B}_{+}\left(R\right)$ is the interior of the hemisphere, ${S}_{+}\left(R\right)$ is the curved surface of the hemisphere where $c={c}_{S}$, and $U\left(R\right)$ is the growth interface where $\partial c/\partial z=\Gamma$. In the center of the growth interface (the origin), $c={c}_{0}$. The subscript “ + ” refers to the upper hemispace.

In order to find $c_{{\max}}$ and $c_{{\min}}$ we aim to solve the steady state diffusion equation,

2 $$ D\nabla^{2} c = 0, $$

with the geometry and the boundary conditions defined in the caption of Fig. 1. In Eq. (2), $\nabla^{2}$ is the Laplacian and $D$ and $c$ are the diffusivity and the concentration of the nanowire species in the liquid particle, respectively. Thus, we consider the following boundary value problem

3 $$\nabla^{2} c = 0 \quad {\text{in}} \; B_{ + } \left( R \right)$$

4 $$c = c_{S} \quad {\text{on}} \; S_{ + } \left( R \right)$$

5 $$\partial c/\partial z = {\Gamma } \quad {\text{on}} \; U\left( R \right),$$

with ${\Gamma } = G/\left( {D{\Omega }} \right)$, where $G$ is the axial growth rate of the nanowire and ${\Omega }$ is the molecular volume of the nanowire species in the solid phase. Since $c$ is, per definition, a harmonic function^11^, its maximum and minimum values are attained on the boundary of the domain. Using the symmetry of the domain it is easy to see that $c_{{\max}} = c_{S}$ and $c_{{\min}} = c\left( 0 \right)$, the latter being the concentration at the origin (the center of the growth interface), which we from now on denote $c_{0}$. The homogeneity coefficient defined in Eq. (1) becomes

6 $$ \chi = \frac{{c_{S} - c_{0} }}{{c_{S} }} . $$

Here we note that both of the concentrations in Eq. (6) are excess concentrations, meaning that the true concentrations are $c_{S} + c_{eq}$ at the surface and $c_{0} + c_{eq}$ is the minimum interface concentration, where $c_{eq}$ is the equilibrium solubility of the species in the metal particle. Most importantly, we also assume that the precursor material supply is efficient enough to ensure a constant surface concentration, $c_{S}$, during some finite time interval, which is longer than the average time it takes for the species to diffuse through the particle.

The problem at hand is to determine an expression for $\chi$ in terms of $R$, ${\Gamma }$, and $c_{S}$. However, with the geometry and boundary conditions according to Fig. 1, Eqs. (3–5) have no analytic solution using the standard technique of separation of variables. So, in order to determine an expression for $c_{0}$, we first solve Eqs. (3–5) for simpler geometries, with the hope of finding a suitable generalization. These analytic calculations are outlined in the next section. Here we only mention the general result, which is that $c_{0} = c_{S} - a{\Gamma }R$, where $a$ is a constant with a value that depends on the geometry of the catalyst particle. Inserting this into Eq. (6), we can express the homogeneity coefficient as a proportionality,

7 $$ \chi = a\frac{{{\Gamma }R}}{{c_{S} }}. $$

In Table 1, we have listed the values of $a$ for the different dimensionalities and geometries considered. For the hemisphere, we show that $a = 1/2$ in a separate section. The readers without interest in the details of our calculations can skip directly to the “Discussion of catalyst particle homogeneity” section without missing anything of essence to our conclusions.

**Table 1:** The proportionality constant, $a$, in Eq. (7) for different model dimensionalities and geometries.

Dimension	Geometry	$a$
1D	Line, length *R*	1
2D	Rectangle, height *R*	0.675
2D	Semicircle	$2/\pi \approx 0.637$
3D	Cylinder, height *R*	0.524
3D	Hemisphere	1/2

## Analytic calculations

Here we show the analytic solutions to Eqs. (3–5) for a few simple geometries and we start with the trivial one dimensional case:

8 $$D\frac{{{\text{d}}^{2} c}}{{{\text{d}}z^{2} }} = 0, \quad c\left( R \right) = c_{S} , \quad \frac{{{\text{d}}c}}{{{\text{dz}}}}\left( 0 \right) = {\Gamma }.$$

The solution to this boundary value problem is $c\left( z \right) = c_{S} - {\Gamma }\left( {R - z} \right)$ and the homogeneity measure in Eq. (7) is given by $\chi = {\Gamma }R/c_{S}$, that is, $a = 1$.

Next we solve Eqs. (3–5) in two dimensions, that is, for a semicircle instead of a hemisphere as in Fig. 1. With boundary conditions corresponding to those in Fig. 1, but with reduced dimension, we get the solution,

9 $$ c\left( {r,\theta } \right) = c_{S} + {\Gamma }r\sin \theta - {\Gamma }R\frac{2}{\pi }\left( {1 + \mathop \sum \limits_{n = 2}^{\infty } \frac{{1 + \left( { - 1} \right)^{n} }}{{1 - n^{2} }}\left( \frac{r}{R} \right)^{n} \cos n\theta } \right), $$

where we have used planar polar coordinates so that $r = \sqrt {x^{2} + z^{2} }$ and $\theta = \arctan \left( {z/x} \right)$. The smallest interface concentration is also in this case found in the origin, $c\left( {0,\theta } \right) = c_{S} - 2{\Gamma }R/\pi$, resulting in the homogeneity measure $\chi = 2{\Gamma }R/\left( {\pi c_{S} } \right)$, that is, $a = 2/\pi$. While these two cases can serve as approximations to the diffusion profile with hemispherical boundary conditions (Fig. 1), they are also interesting in their own right. The one-dimensional case corresponds to VLS film growth^12^ and the two-dimensional case could be relevant for VLS growth of flat, fin- or sail-like structures^13^.

For the sake of completeness we write down the solution to the two dimensional boundary value problem for a rectangular boundary with sides of height $R$ and a top edge of width $2R$. At the sides and the top edge $c = c_{S}$ and at the bottom edge $\partial c/\partial z = {\Gamma }$. With these boundary values, the solution to Eq. (2) is

10 $$ c\left( {x,z} \right) = c_{S} - 2{\Gamma }R\mathop \sum \limits_{n = 0}^{\infty } \frac{{\left( { - 1} \right)^{n} }}{{\lambda_{n}^{2} }}\frac{{\sinh \left( {\lambda_{n} \left[ {1 - z/R} \right]} \right)}}{{\cosh \lambda_{n} }}\cos \left( {\lambda_{n} \frac{x}{R}} \right), $$

where $\lambda_{n} = \left( {n + 1/2} \right)\pi$. The smallest interface concentration is given by $c_{0} = c_{S} - 2{\Gamma }R\mathop \sum \limits_{n = 0}^{\infty } \frac{{\left( { - 1} \right)^{n} }}{{\lambda_{n}^{2} }}\tanh \lambda_{n} \approx c_{S} - 0.675{\Gamma }R$, resulting in $\chi \approx 0.675{\Gamma }R/c_{S}$ ($a \approx 0.675$).

Finally, we generalize the rectangular boundary and solve Eq. (2) in three dimensions with cylindrical boundary conditions. That is, we assume that the catalyst particle is shaped like a cylinder of radius $R$ and height $R$. The concentration at the top circular area and at the side surface is $c_{S}$ and at the bottom circular area (the nanowire growth interface) we have the flux boundary condition $\partial c/\partial z = {\Gamma }$, similar to the previous cases. With these boundary conditions, the solution to Eq. (2) is given by

11 $$ c\left( {r,z} \right) = c_{S} - 2{\Gamma }R\mathop \sum \limits_{n = 1}^{\infty } \frac{{J_{0} \left( {\alpha_{0n} r/R} \right)}}{{\alpha_{0n}^{2} J_{1} \left( {\alpha_{0n} } \right)}}\frac{{\sinh \left( {\alpha_{0n} \left[ {1 - z/R} \right]} \right)}}{{\cosh \alpha_{0n} }}, $$

where $J_{0}$ and $J_{1}$ are the zeroth and first order Bessel functions of the first kind, respectively. The parameter $\alpha_{0n}$ is the *n*th zero of $J_{0}$. The minimum concentration at the growth interface can be calculated as $c_{0} = c_{S} - 2{\Gamma }R\mathop \sum \limits_{n = 1}^{\infty } \frac{{\tanh \alpha_{0n} }}{{\alpha_{0n}^{2} J_{1} \left( {\alpha_{0n} } \right)}} \approx c_{S} - 0.524{\Gamma }R$, resulting in $\chi \approx 0.524{\Gamma }R/c_{S}$ ($a \approx 0.524$).

## Scaling analysis

Since we have seen that the homogeneity index can be written in the form of Eq. (7) for all the investigated cases, we use a scaling approach to show that this proportionality is general and therefore valid for the more realistic geometry in Fig. 1. The problem at hand, Eqs. (3–5), contains a characteristic length scale, $R$, and a characteristic concentration, $c_{S}$. Based on this we introduce the dimensionless variables $\overline{\user2{x}} = R^{ - 1} {\varvec{x}}$ and $u\left( {\overline{\user2{x}}} \right) = c_{S}^{ - 1} c\left( {R\overline{\user2{x}}} \right)$. With these variable changes, Eqs. (3–5) transform into

12 $$\nabla^{2} u = 0 \quad {\text{in}} \; B_{ + } \left( 1 \right)$$

13 $$u = 1 \quad {\text{on}} \; S_{ + } \left( 1 \right)$$

14 $$\partial u/\partial z = {\upgamma } \quad {\text{on}} \; U\left( 1 \right),$$

where we have dropped the overbars and derivatives are taken with respect to the dimensionless variables. The parameter $\gamma$ is given by $\gamma = R{\Gamma }/c_{S}$. In terms of the new variables, the homogeneity coefficient becomes

15 $$ \chi = 1 - u\left( 0 \right). $$

We will now express $\chi$ in terms of $\gamma$, which is the only parameter left in the problem. Let $u_{\gamma }$ denote the solution to Eqs. (12–14) for a given scaled flux $\gamma$. Specifically, if $\gamma = 0$ then $u_{0} \left( {\varvec{x}} \right) = 1$ in $B_{ + } \left( 1 \right)$. Suppose that we have found $u_{1} \left( {\varvec{x}} \right)$, then we can construct $u_{\gamma }$ as

16 $$ u_{\gamma } \left( {\varvec{x}} \right) = \left( {1 - \gamma } \right)u_{0} \left( {\varvec{x}} \right) + \gamma u_{1} \left( {\varvec{x}} \right), $$

which is clearly harmonic and satisfies the boundary conditions. Since it is a solution, it is the unique solution^14^. Inserting $u_{\gamma } \left( 0 \right) = 1 - \gamma \left[ {1 - u_{1} \left( 0 \right)} \right]$ into Eq. (15) we arrive at

17 $$ \chi = \gamma \left[ {1 - u_{1} \left( 0 \right)} \right], $$

which proves Eq. (7), since $\gamma = {\Gamma }R/c_{S}$, and the proportionality constant $a$ can be identified as $a = 1 - u_{1} \left( 0 \right)$.

**Figure 2:**
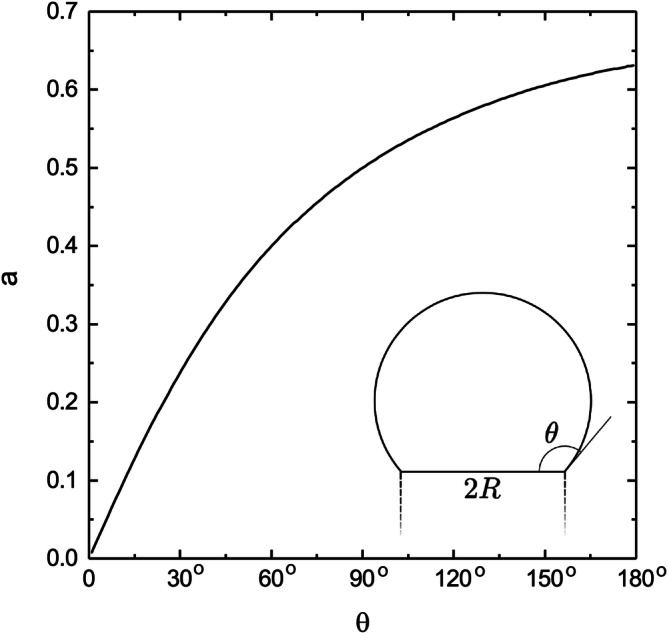
The proportionality constant $a$ as a function of the contact angle $\theta$ as defined in the inset, calculated by finite element modelling and explicit calculation of the homogeneity index according to Eq. (6). Note that in the limit as $\theta \to 180^\circ$, the radius of the largest modelled particle is almost 100 times the wire radius $R$, but $a$ nonetheless remains limited to 0.63.

It is easy to verify that the proportionality in Eq. (17) holds for any reasonable domain if the part of the boundary where the flux $\gamma$ is defined can be described using one characteristic length scale. However, $a$ will be different for differently shaped domains and it can only be analytically calculated in certain special cases, as we have seen. In Fig. 2 we show numeric calculations of the proportionality constant, $a$, which serves as a shape factor, for different values of the contact angle, $\theta$, describing the shape of the liquid metal particle. The value of $a$ remains finite for the full range of $\theta$, with $a$ approaching zero in the small $\theta$ limit and $a \approx 0.63$ as $\theta$ approaches 180°. We also see that $a = 0.5$ at $\theta = 90^\circ$ and in the next section we will show that this is an exact result.

## Homogeneity coefficient for the hemisphere

We now turn to the case of the hemisphere. In order to compute $a$ in this case, the new dependent variable $w\left( {\varvec{x}} \right) = u_{1} \left( {\varvec{x}} \right) - z$ is introduced. Notice that the function $w$ is harmonic and is a solution to the boundary value problem

18 $$ \nabla^{2} w = 0 \quad {\text{in}} \; B_{ + }  \left( 1 \right) $$

19 $$ w = 1 - z \quad {\text{on}} \; S_{ + } \left( 1 \right) $$

20 $$ \partial w/\partial z = 0 \quad {\text{on}} \; U\left( 1 \right). $$

The boundary condition on $U\left( 1 \right)$ is homogeneous, which allows us to symmetrize the problem about the plane $z = 0$. In other words, we seek a solution $\tilde{w}$ to the following Dirichlet problem on the entire unit sphere, $B\left( 1 \right)$ with surface $S\left( 1 \right)$,

21 $$ \nabla^{2} \tilde{w} = 0 \quad {\text{in}} \; B\left( 1 \right) $$

22 $$ \tilde{w} = 1 - \left| z \right| \quad {\text{on}} \; S\left( 1 \right). $$

It is well-known that such a problem has a unique solution $\tilde{w}$, which is infinitely differentiable on $B\left( 1 \right)$ and extends continuously onto the boundary $S\left( 1 \right)$. Furthermore, the reflected function $\tilde{w}\left( {x,y, - z} \right)$ is also a solution to Eqs. (21–22). Since the solution is unique it follows that $\tilde{w}$ is an even function with respect to reflection about the plane $z = 0$ and therefore $\partial \tilde{w}/\partial z = 0$ for $z = 0$. If we define $w$ to be the restriction of $\tilde{w}$ to $B_{ + } \left( 1 \right)$, then $w$ is the desired solution to Eqs. (18–20).

It is clear that $u_{1} \left( 0 \right) = w\left( 0 \right) = \tilde{w}\left( 0 \right)$ and the latter can be computed using the mean value theorem for harmonic functions^11^:

23 $$  \tilde{w}\left( 0 \right) = \frac{1}{{\left| {S\left( 1 \right)} \right|}}\int\limits_{{S\left( 1 \right)}} {\tilde{w}\left(  {\varvec{x}} \right)d\sigma \left(  {\varvec{x}} \right)} ,  $$

where $d\sigma$ is the surface-area measure on $S\left( 1 \right)$ and $\left| {S\left( 1 \right)} \right| = 4\pi$ (the surface-area of the unit sphere). We know that $\tilde{w} = 1 - \left| z \right|$ on $S\left( 1 \right)$ and inserting this in Eq. (23) and using, for instance, spherical coordinates it is a trivial task to compute $\tilde{w}\left( 0 \right) = 1/2$. From this follows that $a = 1 - \tilde{w}\left( 0 \right) = 1/2$ for the hemisphere in Fig. 1.

## Discussion of catalyst particle homogeneity

In this section, we will discuss the homogeneity coefficient, $\chi$, and relate it to nanowire growth experiments, both VLS and vapor–solid–solid (VSS) growth. We will also relate $\chi$ to other dimensionless numbers.

Since the previously outlined analytically solvable cases can serve as approximations to the more realistic problem, the values of $a$ are collected in Table 1, for easy comparison. Here we see that the one-dimensional approximation, Eq. (8), gives an error of a factor of two, which can still be acceptable, given the extreme simplicity of this approximation. The cylinder approximation overestimates $\chi$ by only 5% and the semicircle one with 27%. In the next section we will indeed use the one-dimensional approximation when calculating the time it takes to refill the particle.

Before we can discuss the homogeneity of the catalyst particle we need to estimate the parameters in Eq. (7). Typical growth temperatures for GaAs nanowires are 400–700 °C and the diffusivity of metal atoms in metal solvents are all in the range 10^–9^–10^–8^ m^2^/s, depending on temperature and materials combination^15^. So, for our order of magnitude estimation it will suffice to set $D_{{{\text{Ga}}}} \approx$ 5 × 10^–9^ m^2^/s for the diffusivity of Ga in a liquid Au–Ga alloy. This agrees well with the DFT calculation of the Ga diffusivity in liquid Au, 3 × 10^–9^ m^2^/s, presented in Ref.^16^. We also set the diffusivity of As in the Au–Ga liquid to $D_{{{\text{As}}}} \approx$ 5 × 10^–9^ m^2^/s. This is consistent with the approximation used by Roy and Chhabra^15^,

24 $$ D_{{{\text{AB}}}} = \frac{{d_{{\text{B}}} }}{{d_{{\text{A}}} }}D_{{{\text{BB}}}} , $$

where $D_{{{\text{AB}}}}$ is the diffusivity of solute A in solvent B, $d_{{\text{A}}}$ and $d_{{\text{B}}}$ are the respective atomic diameters, and $D_{{{\text{BB}}}}$ is the self-diffusivity of B. Combining Eq. (24) for the two cases: diffusion of Ga in Au–Ga and diffusion of As in Au–Ga, we can eliminate the atomic diameter and the self-diffusivity of Au. Then, since the van der Waals radii of Ga and As are almost equal ($r_{{{\text{Ga}}}} =$ 1.87 Å and $r_{{{\text{As}}}} =$ 1.85 Å)^17^, the approximation $D_{{{\text{Ga}}}} \approx D_{{{\text{As}}}}$ is justified. Our estimation of $D_{{{\text{As}}}}$ also agrees with the diffusivities of about 4 × 10^–9^ m^2^/s, extracted from liquid phase epitaxy experiments^18,19^ but also measured by Dawson (see Ref.^18^). Next we approximate the surface concentration as $c_{S} = x_{S} \rho$, where $x_{S}$ is the atomic fraction of the nanowire species, and $\rho$ is the atomic density of liquid Au, $\rho =$ 5.3 × 10^28^ m^-3^. The volume of a Ga–As pair in the solid is given by ${\Omega } =$ 4.52 × 10^–29^ m^3^.

**Figure 3:**
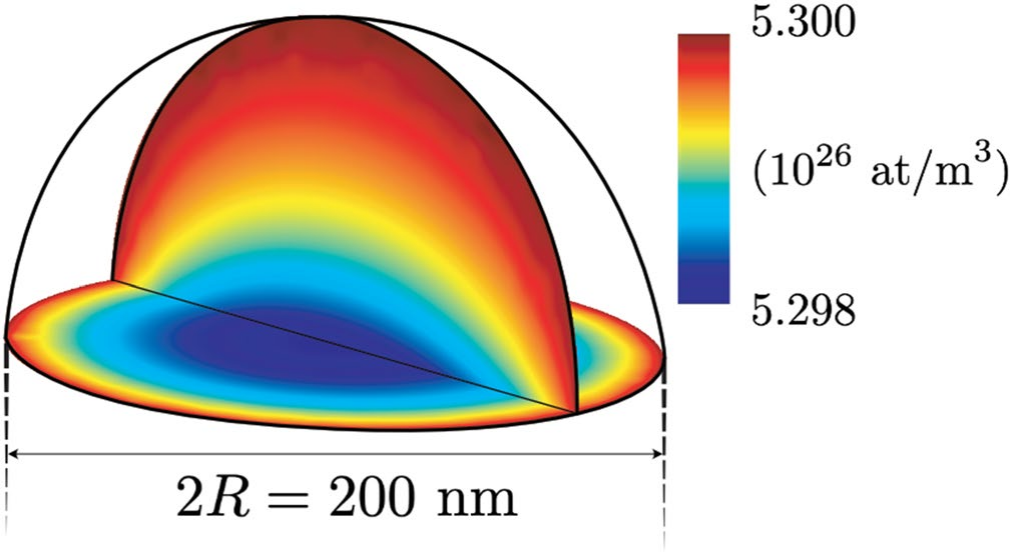
Numeric calculation of the concentration in the catalyst particle for the parameters $D=$ 5 × 10^–9^ m^2^/s, ${c}_{S}=$ 5.3 × 10^26^ m^-3^, $\Omega =$ 4.52 × 10^–29^ m^3^, $G=$ 1 μm/s, $R=$ 100 nm. The color scale indicates the (very small) concentration gradient.

Now we can calculate $\chi$ for the growth of Au-catalyzed GaAs nanowires growing by the VLS mechanism. In order to find an upper limit for $\chi$, we insert the highest axial growth rate that we are aware of, $G\approx$ 1 μm/s, observed for aerotaxy growth^10^. We use a large nanowire radius, $R=$ 100 nm and we let the growth be As limited, with an As concentration at the surface of 1 at%, $x_{{\text{S}}} =$ 0.01. For this set of parameters we get $\chi =$ 4 × 10^–4^, that is, the catalyst particle is very close to being homogeneous also for this extremely high growth rate. This means that it is always safe to assume that the particle is compositionally homogeneous for VLS growth and that the axial growth rate is not limited by diffusion through the liquid catalyst particle. In Fig. 3 we show a numerical calculation of the concentrations in the catalyst particle for this set of parameters. Here it is clearly seen that the variation in concentration is extremely small and that the minimum concentration is at the center of the growth interface.

For the same set of parameters, we can in fact estimate what the growth rate would be if diffusion through the liquid were rate limiting. In this case we set $\chi = 1$ and solve for the growth rate, resulting in the enormous growth rate $G =$ 240 μm/s. It seems unlikely that crystalline nanowires can form with this high growth rate. On the other hand, diffusion through the particle can be rate limiting for VSS growth, where the diffusivity is orders of magnitude smaller. Persson et al.^20^ investigated gold catalyzed growth of GaAs nanowires using chemical beam epitaxy, and concluded that the growth proceeded by the VSS mechanism and was limited by Ga diffusion through the solid catalyst particle. Indeed, using the data from this investigation, we estimate a homogeneity index of $\chi \approx 1$, indicating a strong concentration gradient in the particle, consistent with diffusion controlled growth. On the other hand, Koryakin et al.^21^ have investigated gold catalyzed growth of InAs nanowires at very low temperatures and also in this case, VSS growth was concluded. However, their relatively high diffusivity and their low growth rate led to a very low homogeneity index, $\chi \approx 10^{ - 5}$. That is, the particles seem homogeneous and diffusion through the bulk of the particle cannot be rate limiting. This is not in conflict with the conclusions of Koryakin et al., who found that the growth is limited by diffusion of As along the nanowire–catalyst interface, which limits the nucleation rate of new layers^21^.

So far we have used a steady state model, meaning that the flux into the particle equals the flux out of the particle, which is equivalent to the growth rate. In certain experimental situations this steady state is broken so that the supply of material is smaller than the growth rate. This happens if the total amount of excess material in the seed particle is not large enough to complete one layer, that is if $c_{S} < 2\sqrt 3 /\left( {a_{L}^{2} R} \right)$ for a cylindrical nanowire growing in a {111}-direction with a hemispherical particle, where $a_{L}$ is the lattice constant. This corresponds to $x_{S} < 2\sqrt 3 /\left( {a_{L}^{2} R\rho } \right)$, which evaluates to approximately 0.002 for $R =$ 100 nm and 0.02 for $R =$ 10 nm. Thus, for sufficiently thin wires, the excess amount of material in the catalyst particle will not suffice to complete a layer.

This leads to a situation where the layer nucleates and grows using both the (small) initial supply in the particle and the flux from the vapor phase. When this small initial supply is consumed, the concentration in the particle reaches a certain critical concentration related to a minimum in the Helmholtz free energy^22^, which we here estimate as $c_{eq}$ ($c_{0} = 0$), which in any case should be the lower limit of this concentration. After this the growth rate of the remaining part of the layer is limited by the flux from the vapor phase and will thus proceed slower than initially. For VLS growth, this effect is known as the “stopping effect”^23^. As we will show in the next section, the timescale for diffusion through the liquid is fast enough for steady state diffusion to set in almost immediately after the concentration is changed. Here we also mention that even in the case when the amount of material in the particle is sufficiently large so that there is no “stopping effect”, there can still be some temporal variation of the concentration and thus of the supersaturation, which gives rise to anti-correlation of nucleation events, so called nucleation anti-bunching^24^.

Finally, we discuss $\chi$ in terms of other dimensionless numbers. The Damköhler number is defined as the reaction rate constant divided by the diffusion rate constant, ${\text{Da}} = k_{r} /k_{d}$^25^. If the nanowire growth rate can be described as a pseudo first-order process, the reaction rate constant can be written as $k_{r} = G/\left( {{\Omega }c_{0} } \right)$. The diffusion rate constant is given by $k_{d} = D/\left( {aR} \right)$. This leads to

25 $$ {\text{Da}} = a\frac{GR}{{D{\Omega }c_{0} }}. $$

Using Eq. (6) we substitute $c_{0} = c_{S} \left( {1 - \chi } \right)$ in Eq. (25) to arrive at ${\text{Da}} = \chi /\left( {1 - \chi } \right)$, or

26 $$ \chi = \frac{{{\text{Da}}}}{{1 + {\text{Da}}}}. $$

As the Damköhler number measures the growth rate in comparison to the diffusion rate, this can be a convenient route to estimate the catalyst particle homogeneity. We immediately see that if ${\text{Da}} \ll 1$, which it is for VLS growth, then $\chi \approx {\text{Da}}.$ On the other hand, for materials systems where ${\text{Da}} \gg 1$, $\chi \approx 1$ and the growth is limited by diffusion through the particle.

Another relevant dimensionless number is the mass transfer Biot number, ${\text{Bi}}_{m}$. It is defined as the mass transfer rate at the interface divided by the mass transfer rate in the bulk^26^. Since the interface mass transfer in this system is identical to the growth rate, we have that ${\text{Bi}}_{m} = {\text{Da}}$.

## Diffusion time

In this section we calculate the time it takes to diffuse through the particle and refill it again to some small supersaturation after a stopping event, so that growth can proceed. To calculate this time, we use the one-dimensional approximation and we change the coordinate system as compared to Eq. (8), so that the particle surface is located at $z = 0$ and that the interface is located at $z = R$. The concentration is still the excess concentration (so that $c = 0$ means that the true concentration is $c_{eq}$). Since we will calculate the refill time, we solve the time dependent diffusion equation with a zero flux boundary condition at the interface, emulating a situation where growth has stopped, according to

27 $$\frac{\partial c}{{\partial t}} = D\frac{{\partial^{2} c}}{{\partial z^{2} }}, \quad c\left( {z,0} \right) = 0, \quad c\left( {0,t} \right) = c_{S} , \quad \frac{\partial c}{{\partial z}}\left( {R,t} \right) = 0.$$

In the similar, standard case, no flux boundary condition is imposed and then the solution is given by

28 $$\frac{{c_{S} - c\left( {z,t} \right)}}{{c_{S} }} = {\text{erf}} \, \frac{z}{{2\sqrt {Dt} }},$$

where ${\text{erf}} \, x$ is the error function, defined by ${\text{erf}} \, x = \frac{2}{\sqrt \pi }\mathop \smallint \limits_{0}^{x} \exp \left( { - y^{2} } \right){\text{d}}y$. The solution to the original problem, Eq. (27), can be constructed by summing translated as well as translated and reflected error function solutions according to Eq. (28), so that all conditions in Eq. (27) are met^27^, which leads to

29 $$\frac{{c_{0} - c\left( {\zeta ,r} \right)}}{{c_{0} }} = {\text{erf}} \, \zeta r + \mathop \sum \limits_{n = 0}^{\infty } \left[ {{\text{erf}}\left( { - \zeta r + 2\left( {2n + 1} \right)r} \right) - {\text{erf}}\left( {\zeta r + 2\left( {2n + 1} \right)r} \right)} \right] + \mathop \sum \limits_{n = 1}^{\infty } \left[ {{\text{erf}}\left( {\zeta r + 4nr} \right) - {\text{erf}}\left( { - \zeta r + 4nr} \right)} \right],$$

where $$\zeta = z/R$$ and $$r = R/\left( {2\sqrt {Dt} } \right)$$.

**Figure 4:**
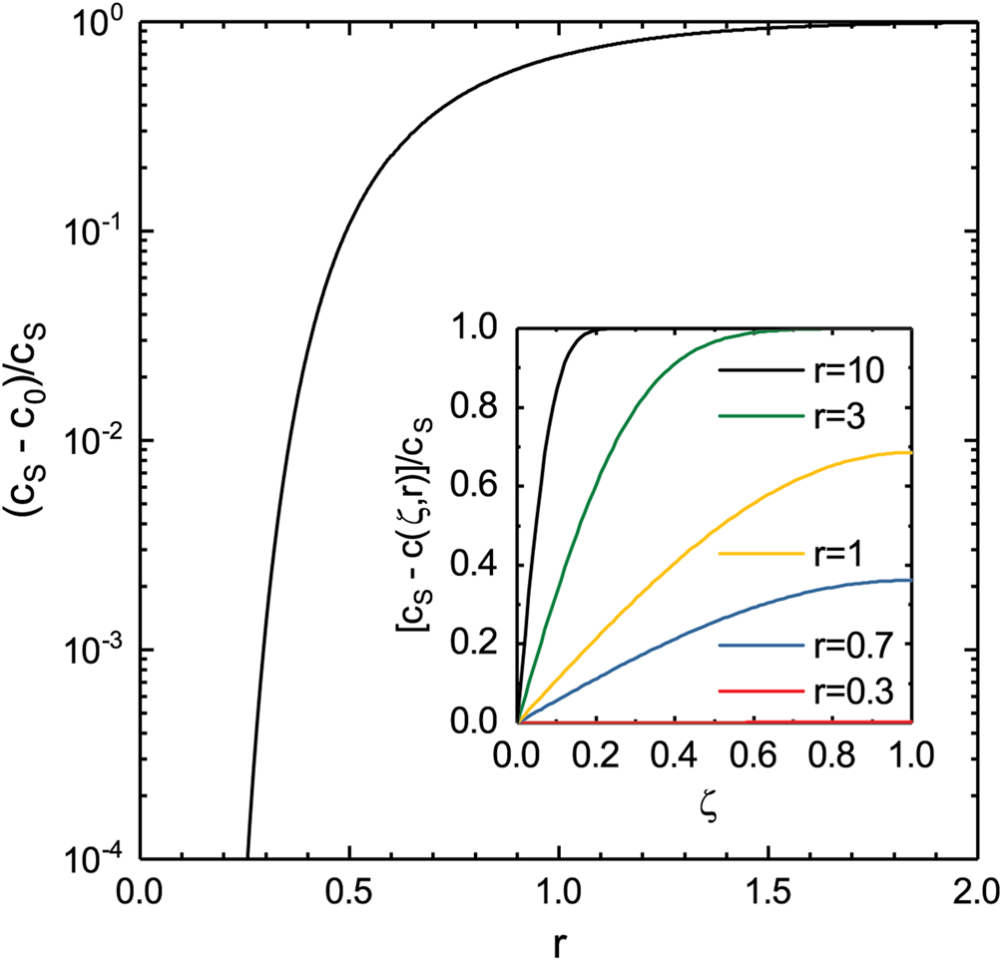
The concentration ratio, $\left({c}_{S}-{c}_{0}\right)/{c}_{S}$, plotted as a function of $r=R/\left(2\sqrt{Dt}\right)$. Note that ${c}_{0}$ is essentially zero for very short times and tends to ${c}_{S}$ in the long time limit. The inset shows the concentration profile for different values of $r$. Note that $\zeta =0$ at the surface of the particle and $\zeta =1$ at the growth interface.

In Fig. 4, we plot $\left( {c_{S} - c_{0} } \right)/c_{S}$, where $c_{0} = c\left( {R,r} \right)$, the concentration at the growth interface, as a function of $r$, our rescaled time variable. We see that as $r$ decreases, or time increases, $c_{0}$ approaches $c_{S}$. The inset shows the concentration profiles, that is $\left( {c_{S} - c} \right)/c_{S}$ as a function of $\zeta$ (where $\zeta = 0$ at the surface and $\zeta = 1$ at the growth interface), for different values of $r$. Using Fig. 4, we can calculate the time it would take for the excess interface concentration to increase from 0 to just below $c_{S}$, that is, the time it takes to make the particle homogeneous. If we for instance choose $c_{0} = 0.99c_{S}$ as a homogeneity criterion (corresponding to $\chi = 0.01$), then for $D =$ 5 × 10^–9^ m^2^/s and $R =$ 100 nm, we get $t =$ 4 µs. If we instead require $c_{0} = 0.999c_{S}$ (or $\chi = 0.001$), we get $t =$ 6 µs for the same choice of parameters. It is interesting to note that this time is independent of the value of $c_{S}$, depending only on the required ratio, $c_{0} /c_{S}$, and for relevant ratios for the homogeneity condition, it depends only weakly on this ratio.

Based on these time estimations we conclude that diffusion through the particle indeed occurs on a much faster time scale than it takes to grow a layer (0.5 ms/layer for Aerotaxy, typically much longer for MOVPE). This implies that after any change in surface concentration, the interface concentration approaches the surface concentration almost instantaneously, which in turn implies that the particle is always compositionally homogeneous, even if its concentration varies with time. Since we have used the one-dimensional approximation we expect the calculated times to be overestimated by a factor of two, which would make the homogeneity argument even stronger, and is in any case accurate enough for an order of magnitude estimation.

## Conclusions

We have investigated the compositional homogeneity of the metal catalyst particle during VLS (vapor–liquid–solid) growth of nanowires. We have introduced a homogeneity measure in the form of a dimensionless number and using steady state diffusion calculations we show that the catalyst particle is homogeneous during VLS growth but that there can be a large concentration gradient through the particle in the case of VSS growth, that is, growth with a solid particle. We have also performed time dependent calculations, which show that the response to a concentration change can be considered instantaneous on the time scale relevant for VLS growth. To conclude, the catalyst particle is homogeneous even if the concentration can vary with time, depending on the growth conditions and the size of the particle. The growth rate is not limited by diffusion through the particle for VLS growth of nanowires.

## Methods

The analytical, steady state solutions to the diffusion equation, Eq. (2), were obtained using the Fourier method, that is, separation of variables. The time dependent solution to the zero flux boundary problem, Eq. (27), was obtained by summing and subtracting translated and reflected as well as only translated error function solutions, which also satisfy the diffusion equation, so that the derivative at the specified location becomes zero and that the surface concentration still has the desired value. This technique has been described by Crank^27^.

The numerical solution to the hemispherical problem was performed with finite element modelling (FEM), using COMSOL Multiphysics software^28^. Equations (3–5) were here solved numerically using a rotated two-dimensional version of the geometry defined in Fig. 1, and a typical result for realistic parameter values is shown in Fig. 3 (solved in 3D for this illustrative plot). The parameter $a$ for varying shapes of the seed particle (spherical segments with contact angle $\theta$) plotted in Fig. 2 was also calculated using FEM in a rotated 2D geometry, with the number of mesh elements at the particle–wire interface constrained to 100 regardless of particle size.
